# A scoping review of interventions to improve blood culture sampling practices in hospital acute care settings

**DOI:** 10.1093/jacamr/dlag009

**Published:** 2026-01-30

**Authors:** Muuna A I Abdi, Deborah Bamber, Carolyn Tarrant

**Affiliations:** Leicester Medical School, University of Leicester, University Road, Leicester, England, LE1 7RH; School of Medical Sciences, University of Leicester, University Road, Leicester, England, LE1 7RH; School of Medical Sciences, University of Leicester, University Road, Leicester, England, LE1 7RH; National Institute for Health and Care Research (NIHR) Greater Manchester Patient Safety Research Collaboration (GM PSRC), University of Manchester, Oxford Road, Manchester, England, M13 9PL

## Abstract

**Background:**

Blood cultures (BCs) are the gold standard investigation for patients with suspected severe infection and sepsis. Yet, BCs are not consistently obtained prior to antibiotic administration, and sampling practices remain suboptimal. Optimizing BC sampling has important benefits, including reducing inappropriate antibiotic use and improving antimicrobial stewardship. Despite advances in sepsis recognition and management, a significant scope remains to improve BC sampling practices. This scoping review aimed to identify evidence on interventions used to improve BC sampling in higher economically developed countries.

**Methods:**

Database searches of MEDLINE, CINAHL, PubMed and BMJ Open Quality were conducted for studies published between January 2015 and January 2025. Included studies were mapped to the Behaviour Change Wheel (BCW) framework.

**Results:**

Searches identified 3746 records; 23 studies met the inclusion criteria, with two additional studies identified through reference screening. In total, 25 studies were analysed, identifying six intervention types. Common interventions included visual prompts, screening tools, education and training programmes and audit-and-feedback mechanisms. These interventions most frequently mapped to the BCW categories of Environmental Restructuring (32%), Education and Training (28%) and Enablement (25%). Outcome measures varied widely, with no consistent metrics used across studies.

**Conclusions:**

This review identified six intervention types used to improve BC sampling practices, with Environmental Restructuring, Education and Training, and Enablement most commonly employed. Interventions were associated with improvements in timely BC collection and reduced contamination rates. However, heterogeneity in outcome measures and gaps in intervention types highlight the need for standardized metrics and more robust evaluations to optimize BC sampling practices across healthcare settings.

## Introduction/background

Bacterial infections present a significant healthcare burden, with serious infections that can lead to sepsis accounting for around 33% of emergency department (ED) admissions.^1^ Blood cultures (BC) are an essential component in the investigation and subsequent management of patients presenting acutely with suspected severe infection and sepsis, enabling identification of the causative organism, guiding appropriate antibiotic choice and improving antimicrobial stewardship (AMS). Overprescribing of antibiotics is a common problem, which contributes to the threat of antimicrobial resistance. Addressing this issue requires the availability of accurate test results, allowing clinicians to confidently establish diagnoses and promptly prescribe appropriate antimicrobial therapy or de-escalate antibiotic treatment.^2,3^ Timely and accurate BC collection enables antibiotics to be reviewed and changed from broad-spectrum to more targeted, effective agents, therefore supporting AMS and efforts to reduce the growth of AMR, and minimizing adverse patient outcomes.^4,5^ Guidelines recommend BCs should be collected before intravenous (IV) antimicrobial therapy is commenced, provided there is no significant delay in starting antibiotics.^6^ Despite this, research suggests that BCs are not consistently taken for patients in ED before treatment with parenteral antibiotics.^2^ The factors influencing variability in BC collection are complex but include patient characteristics and clinical presentation, staff knowledge and attitudes, interpersonal communication, situational factors such as workload and time constraints and organizational policies and practices.^3^

Standards of BC sampling often fall short of best practice guidelines, with factors such as low volume collection, poor sampling technique and user contamination contributing towards void and false positive tests, further delaying appropriate treatment.^6^ Contamination rates reported in the literature differ, though the highest rates are most often observed in the ED; research suggests contributors may include constant staff rotation, competing clinical priorities and the acute nature of patient presentations.^7,8^ BC contamination complicates efficient interpretation, resulting in clinical uncertainty, unnecessarily lengthy hospital stays and increased costs.^9–11^

In England, national guidance has outlined key recommendations for optimizing BC sampling pathways.^1^ Despite this, challenges in the improvement of BC practices remain, such as variability in implementation, infrastructure limitations and gaps in education and awareness.^12^ While examples of successful local improvement exist, achieving consistent and sustainable change across healthcare systems remains challenging. One contributing factor is the inconsistent dissemination of locally conducted quality improvement projects (QIPs) across National Health Service (NHS) trusts.^12^ This lack of data sharing leads to wasted resources in designing and piloting similar interventions without considering modifications based on challenges faced by other organizations. Further, there is a need to ensure that any interventions to improve BC sampling practices are theory-informed. In relation to interventions to improve antibiotic prescribing practice, evidence demonstrates that interventions incorporating effective theory-informed behaviour change techniques are more effective for improving practice.^13^ The case for drawing on social and behavioural science to drive sustainable change in antibiotic prescribing practices has been strongly made, and this applies equally to efforts to improve BC sampling practices.^14^

This scoping review aims to identify the scope of literature around the improvement of BC sampling in higher economically developed countries. The review will systematically classify and describe existing interventions, using the Behaviour Change Wheel (BCW) framework to categorize intervention types. The BCW is a comprehensive behavioural sciences framework developed from the synthesis of 19 different behaviour change frameworks. The integration of the COM-B model (‘Capability’, ‘Opportunity’ and ‘Motivation’ as the main drivers of ‘Behaviour’, respectively) within the BCW explores the facilitators and barriers to specific behaviours, ensuring the causes of poor practice are identified to aid in the design of sustainable behavioural change.^15^ When applied to behaviour change research, evidence-based frameworks provide a systematic approach to classifying and describing interventions.^16^ This review will characterize interventions to improve BC sampling, assess evidence of effectiveness and explore implementation barriers and facilitators, providing valuable insights into the design elements of a successful BC sampling improvement strategy.

### Review question

What is the current extent of evidence on strategies and interventions to improve BC sampling practices, for adults presenting with suspected severe infection and/or sepsis to acute care settings, within higher economically developed countries, and what gaps exist in the literature?

## Methods

This scoping review was conducted in line with the Joanna Briggs Institute (JBI) methodology for scoping reviews^17^ and reported according to the Preferred Reporting Items for Systematic reviews and Meta-Analyses extension for Scoping Reviews (PRISMA-ScR).^18^

### Eligibility criteria

The PCC framework (Population, Concept, Context) was used to develop the eligibility criteria as shown in Table 1.

**Table 1.** Inclusion and exclusion criteria for literature search strategy, classified into PCC criteria

PCC element	Inclusion criteria	Exclusion criteria
Population	Adults (19+ years) Suspected severe infection and/or suspected sepsis	Paediatric and/or neonatal populations No suspected severe infection and/or suspected sepsis
Concept	Interventions to improve blood culture sampling reliability and quality Quality improvement projects to improve blood culture sampling practice	Studies unrelated to quality improvement or interventions to improve blood culture sampling practice Studies focussing on laboratory and/or novel techniques for blood culture analysis
Context	Acute hospital setting Higher economically developed country	Non-acute hospital setting e.g. outpatient clinic Non-higher economically developed country

### Search strategy

Preliminary searches were conducted across selected databases to refine the search strategy and search terms. Databases searched were Ovid MEDLINE, CINAHL, PubMed and BMJ Open Quality [see Table S1 supplementary information (available as Supplementary data at *JAC-AMR* Online)]. Reference and citation lists were screened for further eligible studies. Non-peer-reviewed literature was excluded from the search. Limitations were set for adults aged 19+ (consistent with the parameters of the databases searched), English language and publication date range between January 2015 and January 2025. This review excluded studies published before January 2015 due to significant advances in BC sampling practices and QI methodologies in more recent years. This review included studies that addressed aspects of reliability and quality of BC sampling, including the number and timing of BC sets collected, adequacy of sample volumes, contamination rates and appropriateness of BCs. Studies that investigated BC contamination solely in the context of laboratory techniques and that did not evaluate interventions to improve BC sampling practices were excluded to maintain focus on QI initiatives.

The review focused on higher economically developed countries for comparability, and to develop learning of relevance for a UK context. The design and implementation of interventions to improve BC practices in low-middle income countries are likely to differ significantly, reflecting local challenges and contextual factors such as limited resources and infrastructure. Higher economically developed countries were defined based on the United Nations’ classification for ‘Developed Economies’.^19^

### Source selection

**Figure 1.**
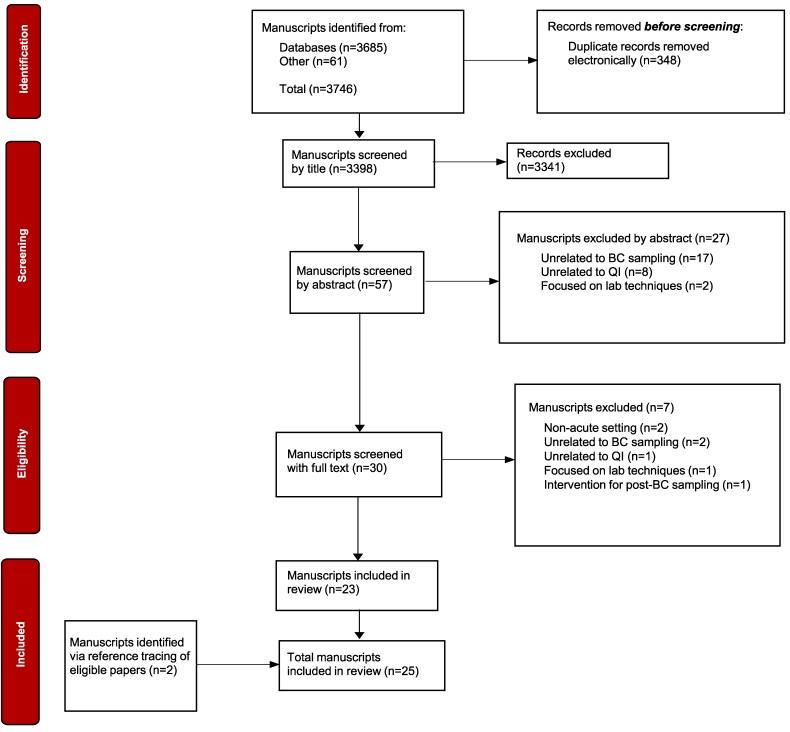
Study selection process outlined via a PRISMA flow diagram.

Quality appraisal of selected eligible studies was not conducted, as per guidance for scoping reviews.^17^ Database search results were exported into EndNote 20 (Clarivate Analytics, PA, USA), where duplicates were electronically removed. Studies were independently screened and assessed for eligibility by title, then abstract initially by the primary reviewer (M.A.). All three reviewers met regularly to compare results, discuss discrepancies and refine the eligibility criteria where necessary. Full texts of potentially eligible articles were then retrieved and assessed independently, with decisions cross-checked and agreed upon collaboratively by the review team for accuracy and quality control. Reasons for exclusion were recorded. Data were extracted on study characteristics, study methodology, intervention types mapped onto the BCW, overall study aims and key BC-related outcome measures. The process of study selection is presented in a PRISMA flow diagram in Figure 1.^18^

## Results

Database searches returned 3746 records and after removing duplicates and screening for eligibility, 23 papers were included, with two further studies identified through reference lists. In total, 25 records were included in this scoping review.^20–44^

### Description of the charted literature

Study details are provided in the supplementary information (supplementary information, Table S2). Most studies were conducted in the USA (*n* = 10), followed by the UK (*n* = 4) with studies taking place in England and Scotland only. Three studies were conducted in Australia, followed by France and Germany, with two studies from each country. The ED was the most common study setting (*n* = 17), with some studies combining the ED with other hospital areas in their research, most often general wards (*n* = 3). Almost all studies employed a quantitative methodology (*n* = 22), with the rest using a mixed methods approach (*n* = 3), incorporating both quantitative outcomes and qualitative data to provide contextual insights. Most studies assessed BC sampling in the context of broader sepsis-related improvement efforts (*n* = 16), with a minority focusing specifically on BC sampling practices (*n* = 9).

**Figure 2.**
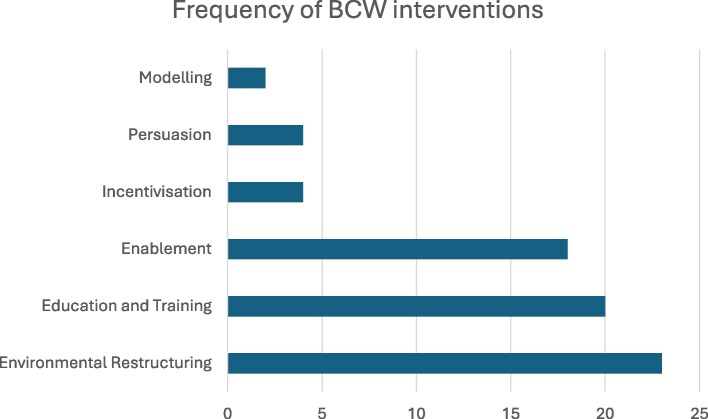
Bar chart representing the frequency of intervention types identified.

In the 25 papers included, 6 intervention types were identified. As shown in Figure 2, a bar chart illustrating the frequency with which individual BCW intervention categories were used across the included studies, the most common interventions related to: ‘Environmental Restructuring’ (32%), ‘Education and Training’ (28%) and ‘Enablement’ (25%). Because studies often employed multiple intervention types, these percentages reflect the proportion of all identified interventions rather than mutually exclusive categories. It was difficult to distinguish between ‘Education’ and ‘Training’ interventions in the studies, since the depth to which these interventions were described varied greatly. Therefore, to improve accuracy and consistency of intervention mapping, ‘Education’ and ‘Training’ were combined as one intervention category (‘Education and Training’) in this review. The outcome measures varied across studies, reflecting the specific aims of each study. The most commonly measured outcome was a compound measure of sepsis bundle compliance, which included BC collection. Other outcome measures reported included 30-day readmissions, median time to antibiotics, appropriateness of antibiotic choice and 28-day mortality.

### Mapping the literature to the BCW theoretical framework

Overall, interventions to improve BC sampling predominantly focused on modifying the clinical environment, supporting staff through education and enabling practice change through audit and feedback. Most interventions were practical and workflow-oriented, such as the use of visual prompts and cues, sepsis algorithms, standardized equipment and performance feedback, often implemented in combination rather than as standalone strategies.

Interventions were classified according to the BCW based on prevalence. The six intervention types identified were: ‘Environmental Restructuring’, ‘Education and Training’, ‘Enablement’, ‘Incentivization’, ‘Persuasion’ and ‘Modelling’. These findings are summarized in Table 2.

**Table 2.** Summary of intervention types, example interventions and reported outcome measures

Behaviour Change Wheel (BCW) intervention type	Common intervention components	Example outcome measures reported
Environmental restructuring	Visual prompts, sepsis algorithms, blood culture (BC) sampling packs, electronic health record (EHR) alerts, ‘sepsis assistants’	Increase in the number of BCs taken prior to antibiotic administration, increase in compliance with sepsis bundle measures, which include BC collection
Education and training	Induction teaching for rotating staff, simulation-based training, workshops	Increase in the rate of BC collection, time to BCs variably reported
Enablement	Audit and feedback, performance reports	Reduction in BC contamination rates, increase in the collection of ‘appropriate’ BCs
Incentivization	Public performance reporting, recognition of staff compliance	Improved adherence to sepsis bundle measures which includes BC collection
Persuasion	Sharing of underperforming cases, reminders	Improved adherence to sepsis bundle measures which includes BC collection
Modelling	Sharing examples of good practice	Reduction in BC contamination rates

The three most common intervention types are explored in detail below: ‘Environmental Restructuring’, ‘Education and Training’ and ‘Enablement’. The least common intervention types are then outlined: ‘Incentivization’, ‘Persuasion’ and ‘Modelling’. The outcomes measured across the body of literature are also considered.

### Environmental restructuring

Environmental restructuring was the most common intervention type, making up 32% of all interventions identified in the studies. The use of visual prompts and cues was most popular (*n* = 16), followed by sepsis algorithms and screening tools (*n* = 10), environmental remodelling (*n* = 7) and sepsis role delegation (*n* = 5).

Passive visual cues included the use of posters, electronic newsletters, pocket cards, photographs and computer screensavers (*n* = 10).^23,25,27,30,33,38,40,42–44^ Staff feedback regarding difficulty remembering sepsis bundle requirements was addressed by attaching a photograph of the bundle components to phlebotomy trolleys in the ED. This provided a passive, constant reminder for staff when treating patients with suspected sepsis.^30^ Informational posters and reference cards detailing the sepsis algorithm were produced and displayed in the ED to reinforce staff awareness of the improvement intervention.^23^ Posters using bold mantras, such as ‘two sets, two sites’, were employed to reinforce correct BC sampling practices following educational sessions.^43^ In contrast, noticeboard educational displays were of limited utility due to inconsistent staff engagement and infrequent updating caused by staffing shortages and competing clinical priorities.^21^

Actionable prompts differ from passive cues in that they require staff acknowledgement and action (*n* = 7).^20,21,26,31,32,39,44^ One study introduced an electronic ED track board displaying real-time patient data (e.g. room number, acuity level, complaint), where a circular icon turned square upon sepsis screening; this visual prompt alerted clinicians to order BCs, aiding timely management.^39^ Another implemented an electronic health record (EHR) pop-up alert that triggered when IV antibiotics were ordered without prior BC collection in 7 days, prompting staff to override, order BCs, or dismiss based on clinical need.^26^

McDonald *et al.*^32^ introduced the Sepsis Now A Priority (SNAP) algorithm, which aimed to provide guidance around the identification, resuscitation and management of sepsis. The Sepsis Early Alert Tool (SEAT), introduced in a different study,^29^ focused on screening sepsis risk factors, categorized into yellow and red criteria, with escalating scores triggering actions such as initiation of the Sepsis-6 bundle or senior clinician review. Sepsis order sets, consisting of a list of standardized investigations available in the ED, were introduced to improve efficiency and increase staff compliance in one study.^39^

Remodelling of environmental factors included standardizing equipment and designating clinical spaces for the care of sepsis patients (*n* = 7).^20,21,25,31,32,39,43^ Equipment standardization included the application of BC sampling packs (*n* = 4)^25,32,39,43^ and BC sampling shelf (*n* = 1).^21^ Bentley *et al.* introduced colour-coded BC sampling shelves (green = preferred closed method; amber/red = non-standard methods) on ED phlebotomy trolleys in their study, allowing staff to deviate when necessary, such as during difficult venous access. These interventions created physical environmental changes to improve workflow and reduce barriers to optimal BC sampling practices.^21^

Various studies created innovative roles for the improvement of sepsis care (*n* = 5).^25,30,36,37,44^ Designated ‘sepsis assistants’ were used within the ED to maintain awareness of the Sepsis-6 bundle by providing advice to staff on initiating treatment and supporting clinical decision-making.^30^ In addition, ‘sepsis champions’ were appointed to support training delivery and act as a point of contact for staff with queries regarding the QI project.^25^ In contrast, other studies repurposed existing roles.^36,37,44^ One study positioned medical students trained in BC taking within the ED to assist nurses by taking the second set of BCs after nurses had taken the first.^44^

### Education and training

In total, 20 studies included Education and Training interventions, and where delivery mode was reported, most were in-person (*n* = 16), with one delivered online (*n* = 1), while several studies did not explicitly specify mode of delivery. The most commonly described approaches were standard lectures (*n* = 12), case-based discussion (*n* = 4) and simulation-based training (*n* = 3); however, not all studies provided sufficient detail to allow categorization of educational format, and some used multiple approaches.

Several studies targeted junior doctors in their education and training interventions (*n* = 5).^20,21,30,35,43^ A mandatory induction training programme was implemented for all junior doctors beginning their ED rotation and delivered by an appointed Lead Sepsis Consultant, covering sepsis diagnostic criteria, management aims and the agreed local guidelines for staff to follow.^20^ Training interventions also targeted rotating junior doctors and addressed topics such as the importance of accurate BC sampling and the consequences of contamination.^21^ In addition, sepsis-focused educational workshops emphasizing the prompt delivery of the Sepsis-6 bundle were delivered and, following positive feedback, repeated during weekly junior doctor teaching sessions.^30^ Brief ‘rapid-fire’ educational sessions on BC sampling technique were also incorporated into resident (junior) doctor orientation programmes.^43^

A tailored sepsis education and training programme was implemented for nurses, healthcare assistants and doctors, covering recognition of sepsis and subsequent management. Staff participated in simulation training with typical and atypical sepsis scenarios.^28^ Simulation-based training was also introduced in another study,^35^ where five sessions were delivered to 120 trainee staff members over a week to allow for practical application of knowledge in a controlled setting.

Video demonstrations of BC sampling techniques were uploaded online to provide staff with easy access.^33,35,43^ Similarly, sepsis algorithms were made available on internal hospital websites to allow continuous staff reference.^41^

Constant staff turnover was a barrier in the implementation of sustainable education and training interventions.^20,21,30,35,41^ Several education and training initiatives targeted junior doctors in an attempt to mitigate this issue.^20,21,30,35^ When the educational sessions were formalized and made mandatory, attendance was high^21,30,35^; however, when additional sessions were designed as drop-ins, there was poor attendance due to conflict with clinical commitments, limiting the sustainability of the intervention.^21^

Constraints in time and resources were reported to be challenges in training implementation, which led to difficulties in the provision of simulation-based training. As a result, training programmes could only be offered to new trainees rather than all staff involved in BC sampling.^35^

### Enablement

In total, 18 studies contained ‘Enablement’ as an intervention, with the most prevalent approaches being feedback (*n* = 16), audit (*n* = 5) and process evaluation (*n* = 2). All 18 studies included either providing feedback (*n* = 10) or collecting feedback (*n* = 5), with two studies using both.

Colour-coded ‘sepsis scorecards’ (green = target met, yellow = within 5 min, red = target missed) were introduced to provide monthly feedback on ED staff performance in adhering to the SNAP sepsis algorithm, reinforcing compliance and motivating improvement.^32^ Written feedback was also generated in one study implementing a diagnostic stewardship intervention, whereby written feedback reports were emailed to frontline staff every 2 months. They found, however, that written feedback reports had a limited impact, as staff engagement with them was inconsistent despite supplemental verbal feedback.^27^ Bidirectional feedback mechanisms were similarly used around newly introduced BC sampling packs, with staff encouraged to provide input and informed when patterns of substandard sampling were identified.^43^

A study aiming to reduce ED BC contamination^21^ sent feedback emails to involved clinicians after each contaminated sample, asking about collection challenges and lessons learned, fostering non-judgmental learning. Iterative feedback was also used to optimize an EHR sepsis-bundle tool: pre-implementation input shaped project design, while ongoing staff input improved tool usability and guided education. Once the tool was launched, compliance metrics and continuous feedback ensured timely adjustments.^42^

Performance was audited during one study to identify gaps between actual and ideal practice. BC events (BCE) were reviewed on a weekly basis and categorized based on their appropriateness against a set BC algorithm. The case reviewers were made up of seven ED clinicians who then created reports detailing the proportion of appropriate versus inappropriate BCEs and their indications. Continual auditing achieved a lower rate of BCEs post-intervention, whilst rates of positive BCs rose, suggesting an improvement in diagnostic stewardship.^41^

### Incentivization, persuasion and modelling

Of the 25 studies included in this review, 8 implemented strategies containing ‘Incentivization’, ‘Persuasion’ and ‘Modelling’. Recognizing achievement was the most common way ‘Incentivization’ was implemented in the studies. Friendly competition between local hospitals was encouraged by publicly publishing performance data, although the exact metrics shared between hospitals were not specified. In doing so, staff engagement improved as well as awareness of the project.^22^ Other studies recognized staff who engaged well with the projects^40,42^; in particular, those who achieved perfect compliance with the sepsis bundle were congratulated for their efforts.

Communicating performance data, particularly when suboptimal, was utilized when implementing ‘Persuasion’ as an intervention type. Underperforming case examples were sent to staff on a monthly basis as active reminders to adhere to the Sepsis-6 bundle requirements.^38^

‘Modelling’ was the least used intervention in this review, making up just 3% of all interventions (*n* = 2). In this context, modelling involved making examples of desired practice visible to staff. Anonymized case summaries were produced and circulated to staff to highlight examples of good practice and key learning points whilst maintaining a no-blame learning environment.^21^ Similarly, examples of high-quality sepsis care were shared with staff to demonstrate expected standards and reinforce desirable clinical behaviours.^24^

### Intervention effectiveness

The majority of included studies reported improvements in their chosen outcome measures; however, outcomes were highly heterogeneous and often embedded within broader sepsis bundle metrics, making it difficult to interpret their specific impact on BC quality. Many studies did not report BC specific measures such as contamination rates, timing of BC collection prior to antibiotics, diagnostic yield or clinical impact (e.g. time to appropriate antibiotics or mortality). As a result, it was not possible to systematically assess the effectiveness of specific intervention types, or combinations of interventions, in terms of their direct effect on improving BC sampling collection. Furthermore, several studies were QI reports using non-randomized, context-specific designs, limiting the strength and generalizability of the evidence. While tentative evidence from the findings presented in the supplementary materials (Table S2) suggests that multimodal interventions combining Education and Training, Environmental Restructuring and Enablement may improve BC collection rates and quality,^20,21,32,33,42^ findings were inconsistent, with at least one study showing no improvement in time to BC collection.^25^

## Discussion

This scoping review examined evidence on interventions to improve BC sampling in adult patients with suspected severe infection and/or sepsis, within acute care settings in higher economically developed countries. Using the BCW framework, six intervention types were mapped, ranked by frequency: Environmental Restructuring, Education and Training, Enablement, Incentivization, Persuasion and Modelling. The most commonly used intervention types were: Environmental Restructuring, Education and Training, Enablement, commonly used in combination, and sometimes with additional intervention types as part of a bundle of interventions.

Michie *et al.*^15^ characterize environmental restructuring as ‘changing the physical or social context’ as a means to reduce barriers to achieving a desired behaviour. This review identified several modes utilized in this intervention category including visual aids, guidelines, algorithms and physical environmental changes. Visual prompts and cues were most commonly used in the studies, likely due to their low cost and ease of implementation. A systematic review assessing the impact of guidelines on health practitioners’ behaviour found passive distribution of guidelines can be effective, sometimes more so than interventions requiring the active engagement of practitioners.^45^ In this scoping review, where guidelines and algorithms were integrated into pre-existing workflow, most often within the EHR, staff engagement with the intervention was high.^26,31,32,39^ Research suggests that in acute care settings, where staff balance competing priorities, the additional cognitive burden caused by excessive alerts and prompts may lead to guideline fatigue.^46,47^ Thus, future QI interventions may benefit from integrating select actionable prompts into pre-existing workflows for improved staff engagement as well as incorporating the use of limited but effective passive visual cues to reinforce knowledge. The use of BC sampling packs has been reported in the literature to optimize BC sampling practices, particularly in reducing BC contamination.^48,49^ Likely, this practice reduces the time and effort taken to gather equipment and limits the likelihood of forgetting a step in the sampling process.

Enablement may be defined as ‘increasing means to increase (an individuals’) capability’ to perform a given behaviour.^15^ In this review, common approaches included feedback and auditing for improvement. The use of feedback within the clinical setting has long been documented as an essential component in the learning process, providing motivation and reinforcing good practice.^50^ Giving and collecting feedback, particularly individualized feedback, can be a good way to engage stakeholders; Marcelino and Shepard^51^ found staff nurses were more interested and receptive when provided with regular informal feedback during their BC contamination reduction QI project. Evidence of how to use feedback effectively exists, but interventions were not always designed in line with this evidence.^52^ When feedback is not given in person, but rather online via reports, there is no guarantee of staff engagement, as found by Fabre *et al.*^27^ Additionally, where there is no established process for giving staff feedback, as reported by Mullane *et al.*,^35^ this can be a costly and timely challenge to implement and maintain. To support the long-term success of improvement initiatives, investment into integrated feedback frameworks will benefit the continuous educational development of staff and encourage engagement with QI efforts long after individual initiatives end.

Education and training interventions identified in this review most often utilized a traditional lecture format, with simulation-based training and case-based discussion also commonly used. The constant changeover of staff was reported as an obstacle in the implementation of improvement initiatives in several studies,^20,21,30,35,41^ with some studies specifically targeting junior doctors in their education and training initiatives in an attempt to mitigate this rotation.^20,21,30,35,43^ Literature suggests that high staff turnover is a barrier in the sustainability of improvement work due to a loss of knowledge and experience related to the intervention, thus requiring re-education of new staff.^53–55^ One study evaluating the sustainability of implementing guidelines targeting nurses found high rates of staff turnover led to a ‘dilution of practices’, resulting in guideline non-adherence.^54^ Regularly repeating education and training sessions strains resources and increases costs. Furthermore, existing staff are often responsible for educating newer staff, contributing to increased workload and time pressures.^53^ It is clear that a constantly fluctuating workforce, coupled with staffing shortages and resource limitations, restricts how well an intervention is sustained long-term. Organizations should seek to optimize the clinical environment to provide practitioners with the opportunity to engage with QI projects, promoting the integration of such interventions into routine clinical practice.^56^

Across our included studies, most interventions were delivered as multimodal bundles combining several behaviour change functions (most often Environmental Restructuring, Education and Training and Enablement). This aligns with evidence from previous studies showing that multimodal strategies—including for instance, in the cases of hand hygiene and the control of healthcare-associated infections—are consistently associated with improved practice and are generally more likely to be effective than single-component approaches.^57,58^ This is a key point of learning for those looking to develop theory- and evidence-based approaches to improving BC sampling practices.

For those planning on developing interventions to improve BC sampling, our review demonstrates that the evidence on which to build these interventions is limited. There is a lack of robust research (e.g. randomized controlled trials) to implement and evaluate the effectiveness of interventions. The problem of identifying effective interventions from the literature is compounded by the lack of consistent outcome measures. For most studies in this scoping review, interventions to improve BC sampling were not the focus and instead, part of a wider set of outcome measures more broadly related to sepsis care. Measures of reliability and quality of BCs were not directly reported. This reflects the realities of clinical practice where BC sampling exists as one component of sepsis management. Yet, where studies do not focus specifically on BC sampling, useful measures specific to BC sampling, such as fill rates and number of sets collected, are not reported, therefore limiting the conclusions that may be drawn regarding the effectiveness of interventions.

We also found that in many studies, both the interventions and the contextual factors were poorly reported, making it difficult, for example, to distinguish clearly between ‘Education’ and ‘Training’ interventions. Inadequate reporting hampers replication, as fidelity to the original design becomes challenging to achieve, and it limits the ability to understand what works, for whom, and in what circumstances. This highlights the importance of researchers using established reporting frameworks such as the TIDieR (Template for Intervention Description and Replication) checklist for describing interventions in detail,^59^ and the SQUIRE (Standards for Quality Improvement Reporting Excellence) guidelines for reporting QIPs.^60^

### Strengths and limitations

Strengths of this work lie in the systematic approach to searching and screening articles, and in the mapping of interventions against the BCW theoretical framework.^15^ In doing so, interventions were classified in a systematic manner to identify commonly used strategies. However, whilst the inclusion of non-UK studies may provide relevant insights into QI efforts, findings may not all be directly comparable to the UK health structure as key differences in resource allocation, staff organization and clinical workflow limit this reviews’ generalizability to the UK context. We only included studies that addressed reliability and quality of BC sampling, and excluded any that solely addressed BC contamination, meaning that additional learning about interventions focused on this specific issue may not have been captured. Reported intervention details varied, especially in ‘Education and Training,’ where unspecified intervention types and implementation hindered cross-study analysis. Furthermore, excluding grey literature may have omitted locally implemented QI interventions, potentially limiting the representativeness of findings for higher economically developed countries.

Our review has highlighted significant gaps in the literature on interventions to optimize BC sampling practices. There is a need for research to develop theory-informed multimodal interventions for BC practices, to establish key measures for assessing the effectiveness of such interventions, and to test their effectiveness using robust study designs. This is critical if we are to improve the extent to which BCs are reliably collected to a high standard, ensuring safe and appropriate treatment for acutely sick patients, and maximizing the opportunities for optimizing antibiotic use and addressing antimicrobial resistance.

## Conclusion

Existing approaches to improving BC sampling practices largely fall within the BCW categories of Environmental Restructuring, Education and Training and Enablement, most commonly delivered through prompts, lectures, training sessions and feedback. However, evidence of their effectiveness is limited. Future research should focus on developing theory-informed, multimodal and sustainable interventions, establishing clear outcome measures and evaluating their impact through robust study designs to improve BC sampling quality, support appropriate treatment and contribute to AMS.

## Supplementary Material

dlag009_Supplementary_Data

## Supplementary data

Tables S1 and S2 are available as Supplementary data at *JAC-AMR* Online.
