# Supplementary material for: A scoping review of interventions to improve blood culture sampling practices in hospital acute care settings
Source: JAC Antimicrob Resist. 2026 Jan 30;8(1):dlag009. doi: 10.1093/jacamr/dlag009 (PMC12856658; doi:10.1093/jacamr/dlag009)
Supplement: dlag009_Supplementary_Data [file dlag009_supplementary_data.zip › 3. Table 1 Supplementary Medline search terms v2.docx]

**Table S1: Ovid MEDLINE search**

| **Search** | **Query** | **Records retrieved** |
| --- | --- | --- |
| 1 | exp Sepsis/ or sepsis.mp. | 232874 |
| 2 | infection.mp. or exp Infections/ | 3885416 |
| 3 | (suspected sepsis or suspected infection or severe infection or suspected severe infection).mp. [mp=title, book title, abstract, original title, name of substance word, subject heading word, floating sub-heading word, keyword heading word, organism supplementary concept word, protocol supplementary concept word, rare disease supplementary concept word, unique identifier, synonyms, population supplementary concept word, anatomy supplementary concept word] | 9377 |
| 4 | 1 or 2 or 3 | 3933372 |
| 5 | emergency service hospital.mp. or exp Emergency Service, Hospital/ | 109606 |
| 6 | emergency medical services.mp. or exp Emergency Medical Services/ | 187256 |
| 7 | hospitalization.mp. or exp Hospitalisation/ | 454951 |
| 8 | patient admission.mp. or exp Patient Admission/ | 27996 |
| 9 | emergency room visits.mp. or exp Emergency Room Visits/ | 4118 |
| 10 | (hospitalisation or acute care or acute admission or acute presentation).mp. [mp=title, book title, abstract, original title, name of substance word, subject heading word, floating sub-heading word, keyword heading word, organism supplementary concept word, protocol supplementary concept word, rare disease supplementary concept word, unique identifier, synonyms, population supplementary concept word, anatomy supplementary concept word] | 59825 |
| 11 | 5 or 6 or 7 or 8 or 9 or 10 | 653338 |
| 12 | exp Quality Assurance, Health Care/ or quality assurance.mp. | 384006 |
| 13 | quality improvement.mp. or exp Quality Improvement/ | 83711 |
| 14 | implementation science.mp. or exp Implementation Science/ | 8916 |
| 15 | (approach or approaches or improve or improving or improvement or service improvement or intervention or implement or strategy or strategies).mp. [mp=title, book title, abstract, original title, name of substance word, subject heading word, floating sub-heading word, keyword heading word, organism supplementary concept word, protocol supplementary concept word, rare disease supplementary concept word, unique identifier, synonyms, population supplementary concept word, anatomy supplementary concept word] | 6869006 |
| 16 | 12 or 13 or 14 or 15 | 7138348 |
| 17 | blood culture.mp. or exp Blood Culture/ | 18313 |
| 18 | (blood culture sampling or blood culture sampling practice*).mp. [mp=title, book title, abstract, original title, name of substance word, subject heading word, floating sub-heading word, keyword heading word, organism supplementary concept word, protocol supplementary concept word, rare disease supplementary concept word, unique identifier, synonyms, population supplementary concept word, anatomy supplementary concept word] | 106 |
| 19 | 17 or 18 | 18313 |
| 20 | 4 and 11 and 16 and 19 | 454 |
| 21 | limit 20 to (English language and yr=“2015 - 2025” and “all adult (19 plus years)”) | 146 |
