# Supplementary material for: A scoping review of interventions to improve blood culture sampling practices in hospital acute care settings
Source: JAC Antimicrob Resist. 2026 Jan 30;8(1):dlag009. doi: 10.1093/jacamr/dlag009 (PMC12856658; doi:10.1093/jacamr/dlag009)
Supplement: dlag009_Supplementary_Data [file dlag009_supplementary_data.zip › 4._Table_2_Supplementary_Study_details_CLEAN.docx]

Supplementary Table 2: Details of included studies.

| **Reference** | **Author** | **Location** | **Overall aim** | **Intervention(s) as mapped onto the BCW** | **Blood culture (BC) related outcomes** |
| --- | --- | --- | --- | --- | --- |
| 20 | Bentley *et al.* 2016 | Scotland | To achieve 75% compliance with the Sepsis-6 bundle in the Emergency Department (ED). | **Education, Training:** Lectures, drop-in sessions, induction training for staff.  **Environmental restructuring:** Introduction of a ‘Sepsis-6 checklist sticker’.  **Enablement:** Regular feedback, surveys and discussion of cases.  **Persuasion:** Monthly compliance rates shared via email and notice board. | A 47.5% improvement in compliance with the Sepsis-6 bundle. |
| 21 | Bentley *et al.* 2016 | Scotland | To achieve a BC contamination rate of below 3% in ED. | **Education, Training:** Drop-in sessions and mandatory induction training.  **Environmental restructuring:** BC sampling shelf attached checklist.  **Enablement:** Regular feedback.  **Persuasion:** Staff updated for every 30 days passed with no contamination, staff congratulated regularly.  **Modelling:** Staff emailed case summaries, with examples of good practice. | BC contamination dropped from 4.61% to 2% within a 12 month period. |
| 23 | Bruce *et al.* 2015 | USA | To understand the impact of a nurse-initiated sepsis protocol in ED. | **Education, Training:** Mandatory online sessions.  **Environmental restructuring:** Posters and reference cards distributed.  **Enablement:** Monitoring of compliance, regular feedback at staff meetings. | Median time to administration of broad-spectrum antibiotics reduced by 27 minutes in the post-protocol group. |
| 24 | Davino *et al.* 2022 | USA | To address gaps in performance, competence and knowledge of sepsis care. | **Education, Training:** Educational meetings, simulation-based training.  **Environmental restructuring:** Point-of-care reminders for clinicians.  **Enablement:** Performance measures, regular feedback.  **Modelling:** Sharing good examples of sepsis care. | No outcomes of interventions measured. |
| 25 | Delawder and Hulton 2020 | USA | To evaluate the impact of implementing a code sepsis team in ED. | **Education, Training:** Quick tip sheets, training.  **Environmental restructuring:** ‘Sepsis champions’, sepsis algorithm, flyers.  **Enablement:** Team feedback and evaluation. | No improvement in time to BCs, despite improvements in all other bundle components. |
| 26 | Dutta *et al.* 2022 | USA | To determine if a decision support intervention improves timely collection of BCs in ED. | **Environmental restructuring:** Pop-up alert prompt to reconsider ordering of antibiotics before BCs completed. | BC collection before intravenous (IV) antibiotics were administered rose from 46.1% pre-intervention to 58.8% post-intervention. |
| 27 | Fabre *et al.* 2020 | USA | To assess the impact of a diagnostic stewardship intervention on the utilisation and appropriateness of BCs in adult medical inpatients. | **Education:** In-person lectures.  **Environmental restructuring:** Algorithm, pockets cards and posters.  **Enablement:** Feedback reports every 2 months via email. | Solitary set BCs reduced from 33% to 26%. Positive BCs increased from 8.1% to 11.5%. |
| 28 | Herron *et al.* 2019 | England | To assess simulation-based training for all staff on sepsis recognition/bundle use. | **Education, Training:** Tailored for each staff group, simulation-based training. | 11% increase in BC collection post intervention. |
| 29 | Idrees *et al.* 2016 | Australia | To evaluate the impact of implementing Sepsis Early Alert Tool on early sepsis recognition and management in ED. | **Environmental restructuring:** Implementation of a screening tool. | Patients who had 2 sets of BCs taken prior to antibiotics increased from 18% to 44%. |
| 30 | Kumar *et al.* 2015 | New Zealand | To assess the effectiveness of Sepsis-6 bundle implementation in the ED. | **Education, Training:** Presentations and workshops.  **Environmental restructuring:** Algorithm, displayed as poster as visual reminder.  ‘Sepsis assistants’ within ED. Photo of bundle components on trolleys.  **Enablement:** Staff feedback and audit. | Increase in BCs taken prior to antibiotics- baseline 33% had BCs of which 78% were before antibiotics versus 6 months post intervention 63% had BCs taken of which 96% were before antibiotics. |
| 31 | Lafon *et al.* 2023 | France | To assess the impact of a dedicated sepsis unit in ED. | **Education, Training:** Sessions for (para)medical staff.  **Environmental restructuring:** 2 rooms designated as sepsis unit, sepsis alert on software system, bundle reminders given. | Higher BC sampling rates post-intervention: phase 1 83%; phase 2 93%. |
| 32 | McDonald *et al.* 2018 | Canada | To standardise the approach to diagnosis and management of sepsis via the implementation of a sepsis algorithm in ED. | **Education, Training:** Via education days, grand rounds and staff meetings.  **Environmental restructuring:** Sepsis algorithm and electronic sepsis indicator on patient tracking board, pre-printed BC order sets.  **Enablement:** Performance data tracked and fed back using a scorecard.  **Incentivisation:** Scorecards were colour coded to highlight positive achievements as well as negative **(Persuasion:)** if targets not met. | Proportion of patients who had BCs taken increased from 95.1% pre-intervention to 99.3% post-intervention. Time to BC draw reduced from 109.8 mins prior, to 73.5 mins post-intervention. |
| 33 | Merien *et al.* 2023 | France | To investigate the impact of a multimodal intervention to improve BCs in ED. | **Education, Training:** Oral presentation, 15-30 min training, educational videos.  **Environmental restructuring:** New local protocol introduced (‘BC train’). Pocket memos distributed to physicians.  **Enablement:** Feedback scheduled before and after each session. | ‘Appropriate’ BC sampling increased from 17.3% pre-intervention to 68.9% post-intervention. |
| 34 | Mohr *et al.* 2021 | USA | To assess the impact of a telemedicine intervention on adherence to sepsis bundle requirements in ED. | **Environmental restructuring:** Implementation of a telemedicine consultation in ED defined as ‘site providers having meaningful audio/video interaction with the hub site, including a clinician interaction’. | 43.2% of patients in the telemedicine group had complete bundle compliance versus 4.5% non-telemedicine group. No difference in adherence to BC collection between groups. |
| 35 | Mullane *et al.* 2024 | Ireland | To implement a combined educational and skin antisepsis intervention aiming to reduce BC contamination rates. | **Education, Training:** Online video demonstration, simulation training.  **Environmental restructuring:** Skin antisepsis intervention, procedure and policy documentation for staff to refer to updated to include it.  **Enablement:** Supervision and feedback. | BC contamination rates dropped from 2.56% pre-intervention to 2.2% post-intervention (not statistically significant). |
| 36 | Nevill *et al.* 2021 | Australia | To examine the impact of the Australasian Triage System (ATS) on sepsis care in ED. | **Environmental restructuring:** Patients were triaged on arrival using the ATS. Designated ED pharmacist assisting with decisions. ‘Sepsis Callout’ system.  **Enablement:** Review and feedback, monthly meetings, audit of treatment times. | Post-intervention, 95% of patients had BCs collected, with 63% collected within 1hr of presenting to ED. |
| 22 | Nsutebu *et al.* 2018 | England | To improve identification and management of sepsis using the Advancing Quality (AQ) Sepsis measure set. | **Education, Training:** Regular learning and networking events between hospitals.  **Enablement:** Feedback of targets and data published.  **Incentivisation:** Publishing of performance against targets by each hospital. | Use of the AQ sepsis measure set, which includes BC collection as part of its protocol, was associated with a 19% reduction in readmission within 30 days. |
| 37 | Rothe *et al.* 2019 | Germany | To evaluate the impact of the implementation of a diagnostic stewardship intervention strategy to improve BCs in ED. | **Education, Training:** Regular sessions, team meetings.  **Environmental restructuring:** Development of local guidelines for staff to follow, full time infectious disease resident in ED for supervision of implementation. | An increase in the number of BCs per person from 26.5% to 63.0% post-intervention. An improved rate of true bacteraemia without any increase in diagnostic yield (1.8% versus 2.5%). |
| 38 | Schwarzkopf *et al.* 2022 | Germany | To assess the effect of a multifaceted educational intervention in improving guideline adherence. | **Education:** Standard lectures and newsletters.  **Environmental restructuring:** Pocket cards, flyers and posters distributed as passive reminders.  **Persuasion:** Suboptimal case examples circulated to staff monthly.  **Enablement:** Audit, quarterly quality reports and monthly feedback of case examples. | The proportion of BCs taken before starting antibiotics did not change significantly between groups. Although more patients had x2 BCs taken post-intervention, this difference was not significant. |
| 39 | Shah *et al.* 2018 | USA | To evaluate the impact of a sepsis screening tool implemented in ED on 3-hour bundle compliance in sepsis patients. | **Environmental restructuring:** Implementation of a sepsis screening tool and electronic tracking board in ED, availability of sepsis order set. | A statistically significant increase in the proportion of patients receiving antibiotics within 3hrs (58.6% versus 89.5%). |
| 40 | Sreeramoju *et al.* 2021 | USA | To implement a 5 year hospital wide initiative with the goal of reducing healthcare associated infection and sepsis mortality. | **Education, Training:** Biweekly lunch and learn sessions.  **Enablement:** Regular feedback, audit and review.  **Environmental restructuring:** Sepsis Early Warning Scores, best practice alerts, screensavers.  **Incentivisation:** Recognition for staff engaging in best practice care. | Adherence to overall sepsis bundle requirements increased from 14% pre-intervention to 34% post-intervention. |
| 41 | Theophanous *et al.* 2024 | USA | To explore impact of a BC algorithm in ED on diagnostic stewardship (appropriate BC ordering and utilisation). | **Education:** Presentations, paper handouts, email.  **Environmental restructuring:** Electronic Health Record (EHR) premade order sets were adjusted, existing sepsis algorithm modified.  **Enablement:** Audit and feedback. | Post-intervention BC events were lower whilst rates of positive BCs increased from 9.6% to 10.6%. |
| 44 | van Daalen *et al.* 2020 | The Netherlands | To utilise a healthcare failure model to optimise the process of BC sampling. | **Education:** 5 lessons focussed on correct sampling technique for ED nurses.  **Environmental restructuring:** Medical students in ED as BC takers, fixing of electronic system for ordering of BC sets.  **Enablement:** Regular emails and monthly newsletter. | Failing to collect a second set of BCs had the highest priority for improvement. Modifying the electronic medical system to allow ordering of 2 sets had the biggest impact on improvement. |
| 42 | Warstadt *et al.* 2022 | USA | To develop a multidisciplinary quality improvement initiative focussed on training on the use of the EHR tool. | **Education:** Nurse-focussed education.  **Environmental restructuring:** Sepsis care reminders posted on all ED computers.  **Enablement:** Nurse, physician and physician associate group and individual feedback.  **Incentivisation:** Positive feedback when excellent bundle care is achieved. | BCs taken before antibiotics increased from 68.5% pre-intervention to 95.1% post-intervention. |
| 43 | Williams *et al.* 2024 | Australia | To explore the impact of an intervention to improve the quality of BCs taken in ED. | **Education, Training:** Small group formal and informal sessions, education videos.  **Environmental restructuring:** Sampling packs, posters detailing correct technique.  **Enablement:** Feedback regarding sampling packs encouraged, real time feedback for staff. | Rates of single set collection reduced from 56.2% pre-intervention to 22.8% post-intervention. Underfilled bottle rates dropped and were sustained 18 weeks post-intervention. |
